# Unprecedented genetic variability of PFam54 paralogs among Eurasian Lyme borreliosis‐causing spirochetes

**DOI:** 10.1002/ece3.11397

**Published:** 2024-05-21

**Authors:** Janna Wülbern, Laura Windorfer, Kozue Sato, Minoru Nakao, Sabrina Hepner, Gabriele Margos, Volker Fingerle, Hiroki Kawabata, Noémie S. Becker, Peter Kraiczy, Robert E. Rollins

**Affiliations:** ^1^ Evolutionary Ecology and Genetics, Faculty of Biology Christian‐Albrechts‐Universität Zu Kiel Kiel Germany; ^2^ Terrestrial Ecology Research Group, Department of Life Science Systems, School of Life Sciences Technical University of Munich Freising Germany; ^3^ Disrupt.Design Lab, Faculty of Architecture and Town Planning, Segoe Building Technion – Israel Institute of Technology Technion City Israel; ^4^ Department of Bacteriology I National Institute for Infectious Disease Tokyo Japan; ^5^ Department of Parasitology Asahikawa Medical University Asahikawa Japan; ^6^ German National Reference Center for Borrelia, Bavarian Health and Food Safety Authority Oberschleissheim Germany; ^7^ Division of Evolutionary Biology, Faculty of Biology LMU Munich Planegg‐Martinsried Germany; ^8^ Institute of Medical Microbiology and Infection Control, University Hospital of Frankfurt Goethe University Frankfurt Frankfurt Germany; ^9^ Institute of Avian Research “Vogelwarte Helgoland” Wilhelmshaven Germany

**Keywords:** *Borrelia burgdorferi* sensu lato, gene evolution, host‐pathogen interactions, Lyme borreliosis, PFam54 gene array, spirochetes

## Abstract

Lyme borreliosis (LB) is the most common vector‐borne disease in the Northern Hemisphere caused by spirochetes belonging to the *Borrelia burgdorferi* sensu lato (*Bb*sl) complex. *Borrelia* spirochetes circulate in obligatory transmission cycles between tick vectors and different vertebrate hosts. To successfully complete this complex transmission cycle, *Bb*sl encodes for an arsenal of proteins including the PFam54 protein family with known, or proposed, influences to reservoir host and/or vector adaptation. Even so, only fragmentary information is available regarding the naturally occurring level of variation in the PFam54 gene array especially in relation to Eurasian‐distributed species. Utilizing whole genome data from isolates (*n* = 141) originated from three major LB‐causing *Borrelia* species across Eurasia (*B. afzelii*, *B. bavariensis*, and *B. garinii*), we aimed to characterize the diversity of the PFam54 gene array in these isolates to facilitate understanding the evolution of PFam54 paralogs on an intra‐ and interspecies level. We found an extraordinarily high level of variation in the PFam54 gene array with 39 PFam54 paralogs belonging to 23 orthologous groups including five novel paralogs. Even so, the gene array appears to have remained fairly stable over the evolutionary history of the studied *Borrelia* species. Interestingly, genes outside Clade IV, which contains genes encoding for proteins associated with *Borrelia* pathogenesis, more frequently displayed signatures of diversifying selection between clades that differ in hypothesized vector or host species. This could suggest that non‐Clade IV paralogs play a more important role in host and/or vector adaptation than previously expected, which would require future lab‐based studies to validate.

## Graphical abstract

Utilizing whole genome sequencing data for 141 Eurasian distributed *Borrelia* isolates, we show that the PFam54 gene array is highly variable among and within *Borrelia* species, including the absence of genes of known function during infection. Further analysis highlighted though that understudied paralogs most likely are important to completing the *Borrelia* life cycle, although with a yet unknown function, requiring further research. Taken together, the results display the importance of studying individual variation in *Borrelia* species from a population genetics perspective to provide novel hypotheses regarding *Borrelia* or vector‐borne pathogen evolution.

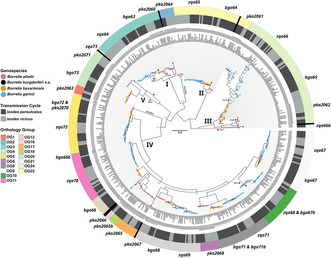

## 1 INTRODUCTION

Lyme borreliosis (LB) is the most common vector‐borne disease in the Northern Hemisphere (Stanek et al., 2011; Steere et al., 2016). This disease is caused by spirochetes belonging to the *Borrelia burgdorferi* sensu lato (*Bb*sl) species complex and are maintained naturally in complex enzootic transmission cycles between ixodid ticks and various vertebrate hosts (Kurtenbach et al., 2006; Stanek et al., 2011). *Borrelia* possess a range of proteins encoded by genes located on a fragmented genome allowing spirochetes to interact with different environmental conditions in tick vectors and vertebrate hosts. The borrelial genome consists of a highly conserved, linear chromosome and up to 20 circular and linear plasmids (Fraser et al., 1997; Kraiczy, 2016; Schwartz et al., 2021). Although much research has gone into understanding the pathogenic processes in humans, many open questions remain regarding the impact of naturally occurring genetic variation in LB spirochetes on underlying molecular infection mechanisms especially in relation to natural transmission cycles.

To establish an infection, *Bb*sl must evade host immune responses including complement as an important pillar of innate immunity. This may be either indirectly through the acquisition of complement regulators or directly through interactions with different complement components (Coburn et al., 2021; Dulipati et al., 2020; Kraiczy & Stevenson, 2013; Lin, Diuk‐Wasser, et al., 2020; Lin, Frye, et al., 2020; Skare & Garcia, 2020). The complement system consists of three distinct pathways (classical, lectin, and alternative) which all converge to the cleavage of the central component C3 to form activated C3b (Atkinson et al., 2019). Host cells control excessive complement damage by utilizing membrane‐bound or fluid‐phase regulator proteins (Atkinson et al., 2019) which can terminate the complement cascade at different activation levels to protect self‐cells from damage (Atkinson et al., 2019). Lyme borreliosis spirochetes produce diverse outer surface proteins that bind distinct host complement components resulting in complement inactivation (Caine et al., 2017; Coburn et al., 2021; Garcia et al., 2016; Kraiczy, 2016; Skare & Garcia, 2020; Xie et al., 2019). One well‐studied protein belonging to the paralogous protein family PFam54 Clade IV, CspA (*bba68*), is capable of binding the fluid‐phase complement regulatory proteins Factor H and Factor H‐like protein 1 (FHL‐1) (Hart et al., 2018; Kraiczy, 2016). Members of the PFam54 are encoded by genes predominantly arranged in a multi‐gene array located at the 5´‐terminal end of the linear plasmid 54 (lp54) in the majority of *Bb*sl isolates studied (Casjens et al., 2012; Hart et al., 2018; Rollins et al., 2022; Schwartz et al., 2021; Wywial et al., 2009). The PFam54 gene array can be separated into five major Clades where Clades I, II, III, and V share one‐to‐one orthology among the *Bb*sl species studied to date (Wywial et al., 2009). Clade IV, however, contains a variable number of paralogs and many species display unique Clade IV paralogs not found in other *Bb*sl species (Wywial et al., 2009). Furthermore, gene array members have been linked to specific host adaptations as well as being differentially expressed during tick and host infection (Caimano et al., 2019; Hart et al., 2018, 2021; Iyer et al., 2015) placing them as suitable candidate genes for study in relation to host and/or vector adaptation. However, the majority of genes present in the *Bb*sl genome, including the PFam54 gene array, are unique to *Bb*sl spirochetes reducing the ability to deduce potential function through comparison to other studied bacterial species (Schwartz et al., 2021). This is where comparative, population genomics can be leveraged to guide future studies through comparing within and among *Bb*sl species which differ in proposed host and/or vector adaptation.

Currently, three major *Borrelia* species act as LB‐causing agents across Eurasia namely *B. afzelii*, *B. bavariensis*, and *B. garinii* (Kurtenbach et al., 2006; Radolf et al., 2012; Stanek et al., 2011; Steere et al., 2016). All three *Borrelia* species share an Asian origin and have expanded into at least two tick transmission cycles (*I. persulcatus*, *I. ricinus*) with this expansion being adaptive toward colonizing a new tick vector (*I. ricinus*) in *B. bavariensis* (Becker et al., 2020; Gatzmann et al., 2015; Margos et al., 2019; Rollins et al., 2023). In both transmission cycles, these *Borrelia* species additionally utilize variable reservoir hosts either infecting rodent (*B. afzelii*, *B. bavariensis*) or avian (*B. garinii*) species (Comstedt et al., 2011; Kurtenbach et al., 2006; Margos et al., 2009; Munro et al., 2017). In the case of *B. bavariensis* and *B. garinii*, these species are sister taxa suggesting a host switch between rodent and avian reservoir hosts has occurred at some point during their evolutionary history (Becker et al., 2016; Margos et al., 2019). Selection for traits such as these can lead toward signatures of adaptation in the genome of these species which can provide information on what genes may be involved in specific phenotypes (Nielsen & Slatkin, 2013). As members of the PFam54 gene array encode proteins with known involvement toward host adaptation (Hart et al., 2018, 2021), we elected to focus on the evolutionary history of this gene family to determine if other genes present in the array may play a yet unknown role in *Bb*sl pathogenesis. Previous work on the diversity of the PFam54 gene array analyzed very few samples and, in the case of Eurasian distributed species, individual isolates (Wywial et al., 2009) or specific PFam54 clades have been studied (Hart et al., 2021). To determine what roles the PFam54 gene members may play in adaptation within Eurasian LB‐causing *Bb*sl spirochetes, we aimed to utilize recently published, whole genome sequencing data from 136 Eurasian *Borrelia* isolates (Rollins et al., 2023) along with published reference genomes (*n* = 5) so as to (1) characterize the diversity of the PFam54 gene array in these isolates to determine the naturally occurring level of genetic variation, (2) robustly reconstruct the phylogeny of the gene family and assign each gene to an orthologous group so as to enable (3) the search for signatures of diversifying selection within the reconstructed phylogeny in relation to different hypothesized reservoir hosts (rodent vs. bird) or tick vectors (*I. ricinus* vs. *I. persulcatus*). Altogether, these findings have the potential to open the door for novel directions in future research to understand how *Bb*sl species have adapted to novel host and vector species.

## 2 METHODS

### 2.1 Isolates and reconstruction of lp54 sequences

In this study, we aimed to determine the level of variation along the PFam54 gene array in *B. afzelii*, *B. bavariensis*, and *B. garinii*. For reconstruction of the lp54 sequences, on which the PFam54 gene array is located, we utilized the isolate library described in Rollins et al. (2023) containing MiSeq sequencing data for 136 Eurasian *Borrelia* isolates: *B. afzelii* (total *n* = 33, Asian *n* = 20, European *n* = 13), *B. bavariensis* (total *n* = 46, Asian *n* = 27, European *n* = 19), and *B. garinii* (total *n* = 57, Asian *n* = 25; European *n* = 32). Asian or European here refers to an isolate hypothesized to be maintained either in the *I. persulcatus* or *I. ricinus* transmission cycle, respectively, based on the geographic origin of isolation. Future analysis will be required though to determine each isolate's capacity to transmit through these tick species specifically.

Sequences of the linear plasmid lp54 were assembled based on the mapping protocol outlined previously (Becker et al., 2020; Rollins et al., 2023). In brief, Illumina reads (paired‐end reads of 250 bp) were first trimmed for Illumina MiSeq adapter sequences using Trimmomatic v.0.38 (Bolger et al., 2014) before being assembled using SPAdes v.3.13.0 (Bankevich et al., 2012). SPAdes contigs were then mapped to reference lp54 sequences (PacBio Sequences: PBi, A104S, NT24, PHei, PBr, and NT31 (Margos et al., 2018; Rollins et al., 2023); GenBank: PKo (CP002950.1), K78 (CP009059.1), and ACA‐1 (CP001247)) using NUCmer v.3.23 from the package MUMmer (Delcher et al., 2002; Kurtz et al., 2004). Lp54 identity was confirmed by searching for plasmid partitioning genes belonging to PFam32, 49, 50, and 57.62 families using BLAST v.2.8.1 (Altschul et al., 1990; Camacho et al., 2009) (algorithm: *blastn*) as outlined in (Becker et al., 2020; Rollins et al., 2023). Final lp54 sequences were determined in one of two ways: (1) a complete contig covering the full reference sequence and containing one or more of the partitioning genes mentioned above was taken without modification as the complete lp54 sequence, or (2) the lp54 was fragmented over two or more contigs for which the best reference (highest percent identity, highest overall coverage, and fewest structural variations) was used to guide reconstruction as outlined in (Becker et al., 2020). In this way, 136 lp54 sequences were reconstructed from NGS data to which the GenBank references used for mapping (*n* = 3) and additional GenBank lp54 sequences (*n* = 2; *B. bavariensis*, BgVir, CP003202.1; *B. garinii*, Far04, CP001318.1) were included in all future analyses bringing the total sample set analyzed to 141 sequences.

### 2.2 Identification and characterization of PFam54 gene array

Sequences for lp54‐located PFam54 paralogs described in (Wywial et al., 2009) for *B. afzelii* PKo (*pko2060*‐*pko2071*), *B. bavariensis* PBi (*bga63‐bga73*), *B. garinii* ZQ1 (*zqa66‐zqa73*), and *B. burgdorferi* B31 (*bba64‐bba66*, *bba68‐70*, *bba73*) were downloaded from GenBank (Accession Numbers: PKo, CP002950.1; PBi, CP000015.1; ZQ1, AJ786369.1; B31, AE000790.2) and used as queries (Note: GenBank and (Wywial et al., 2009) designations are not identical but numbering is). We used BLAST v.2.8.1 (Altschul et al., 1990; Camacho et al., 2009) (algorithm: *blastn*) to search for paralogs described above. A BLAST approach was chosen to conform to previous work to describe the PFam54 family (Wywial et al., 2009). Blast hits shorter than 500 bp and with a percentage identity lower than 80% compared to the reference were not considered paralogous. Further BLAST hits were removed if they were overlapping with regions already designated as a different paralog. BLAST hit lists were manually checked for intergenic regions >1000 bp, which were extracted and scanned for open reading frames in Aliview v.1.28 (Larsson, 2014). Final PFam54 gene sequences were compared against their own reference lp54 sequences and used to produce final gene lists. Gene assignments were checked through phylogenetic reconstruction (see below). Final gene lists were used to define PFam54 gene array architecture types (i.e., structure of gene array taking gene order, content, and gene/intergenic space length into account). An architecture type was defined based on the following rules: (1) same paralogs present in the same order, (2) gene length does not differ by more than ±50 bp, (3) intergenic spaces do not differ by more than ±100 bp.

### 2.3 Phylogenetic reconstruction and selection testing

To understand the evolution of the PFam54 gene family, a robust reconstruction of the evolutionary history of the orthologous genes in all borrelial isolates analyzed was required. We opted for phylogenetic reconstruction taking codon variation into account, which should capture the evolution of protein coding sequences better than simple nuclear substitution models (Goldman & Yang, 1994; Muse & Gaut, 1994). Only unique PFam54 sequences (*n* = 524 of 1308 sequences) were used for phylogenetic reconstruction. Final stop codons were removed before aligning as amino acids using MUSCLE v3.8.425 (Edgar, 2004a, 2004b) implemented in Aliview v1.28 (Larsson, 2014). Phylogenetic reconstruction was run in MrBayes v.3.2.6 (Huelsenbeck & Ronquist, 2001; Ronquist et al., 2012) with the following parameters: ploidy = haploid, codon substitution model with inverse gamma‐distributed rate variation, genetic‐code = universal, and assuming equal site selection (ω) (Goldman & Yang, 1994; Muse & Gaut, 1994). Three independent runs were launched for 50 million generations each. Convergence was checked with Tracer v.1.7.1 (Rambaut et al., 2018). Consensus trees were built using the *sumt* command from MrBayes using a respective burn‐in of 25%. Convergence to a single topology in all three independent runs was checked manually in FigTree v.1.4.4 (http://tree.bio.ed.ac.uk/software/figtree/). Gene orthology of individual paralogs (i.e., orthology groups) was based on the monophyletic clustering of individual gene copies either for single or multiple *Borrelia* species.

In addition to reconstructing the gene phylogeny, we further aimed to test for instances of diversifying selection. As signals of positive selection can often be hidden by negative selected sites (Zhang et al., 2005) and selection coefficients (*ω*_n_) could vary along different branches within the reconstructed gene phylogeny, we chose a model for selection inference that allows for variation in selection pressure between branches and sites. We tested for instances of diversifying selection along the tree reconstructed with the codon model in MrBayes using aBSREL v2.2 (Smith et al., 2015) from the HyPhy package (https://www.hyphy.org/) using the universal genetic code and not allowing for multiple hits. Positive selection on a branch is indicated by a significant result of the likelihood ratio test (LRT) performed by aBSREL after multiple testing corrections by the Holm–Bonferroni sequential rejection procedure. Four different kinds of branches were tested: branches separating species that utilize different vertebrate hosts (bird vs. rodent), branches separating isolates hypothesized to be maintained in transmission cycles involving different tick vectors (*I. persulcatus*, *I. ricinus*), genes that appear to have arisen through recombination based on our analysis (e.g., *bga67b*), or genes known to encode proteins that have been shown to provide protection from host‐immune mediated killing (*zqa68*, *bga66 bga71*, *pko2068*) (Hammerschmidt et al., 2014, 2016; Hart et al., 2018, 2021; Kraiczy, 2016). This resulted in a total of 44 branches being tested.

### 2.4 Determining gene gain and loss events along the PFam54 gene array

Gene gain and loss events were determined by reconstructing the phylogeny of the entire lp54 after removing any recombining region following the four‐gamete condition test as outlined in (Gatzmann et al., 2015; Rollins et al., 2023). Full, corrected lp54 sequences were aligned using MAFFT v.7.407 (Katoh et al., 2002; Katoh & Standley, 2013). Phylogenetic reconstruction was performed in MrBayes v.3.2.6 (Huelsenbeck & Ronquist, 2001; Ronquist et al., 2012) with ploidy set to haploid and a GTR (Tavaré, 1986) substitution model with inverse gamma‐distributed rate variation. Three independent runs were launched and ran for 10 million generations. Convergence was checked with Tracer v. 1.7.1 (Rambaut et al., 2018). Consensus trees were built using the *sumt* command from MrBayes using a respective burn‐in of 25%. Convergence to a single topology in all three independent runs was checked manually in FigTree v.1.4.4 (http://tree.bio.ed.ac.uk/software/figtree/). Gene gain or losses were then mapped onto the final lp54 tree using the maximum parsimony principle.

### 2.5 Statistical analysis

Clustering based on a binary matrix of orthology group (OG) presence or absence was run using classical multidimensional scaling (MDS) run with the *cmdscale* function using the base R package on a distance matrix calculated from the binary presence/absence plasmid data per isolate. Saturation curves were produced by randomly sampling architecture types 50 times for each number of isolates less than or equal to the total number of isolates present from a single species in each transmission cycle from all possible combinations of architecture types. The number of unique architecture types was determined per each random sample. The mean and standard deviation were calculated for each sampling event in R v.3.5.2 (R Core Team, 2019).

## 3 RESULTS

**FIGURE 1:**
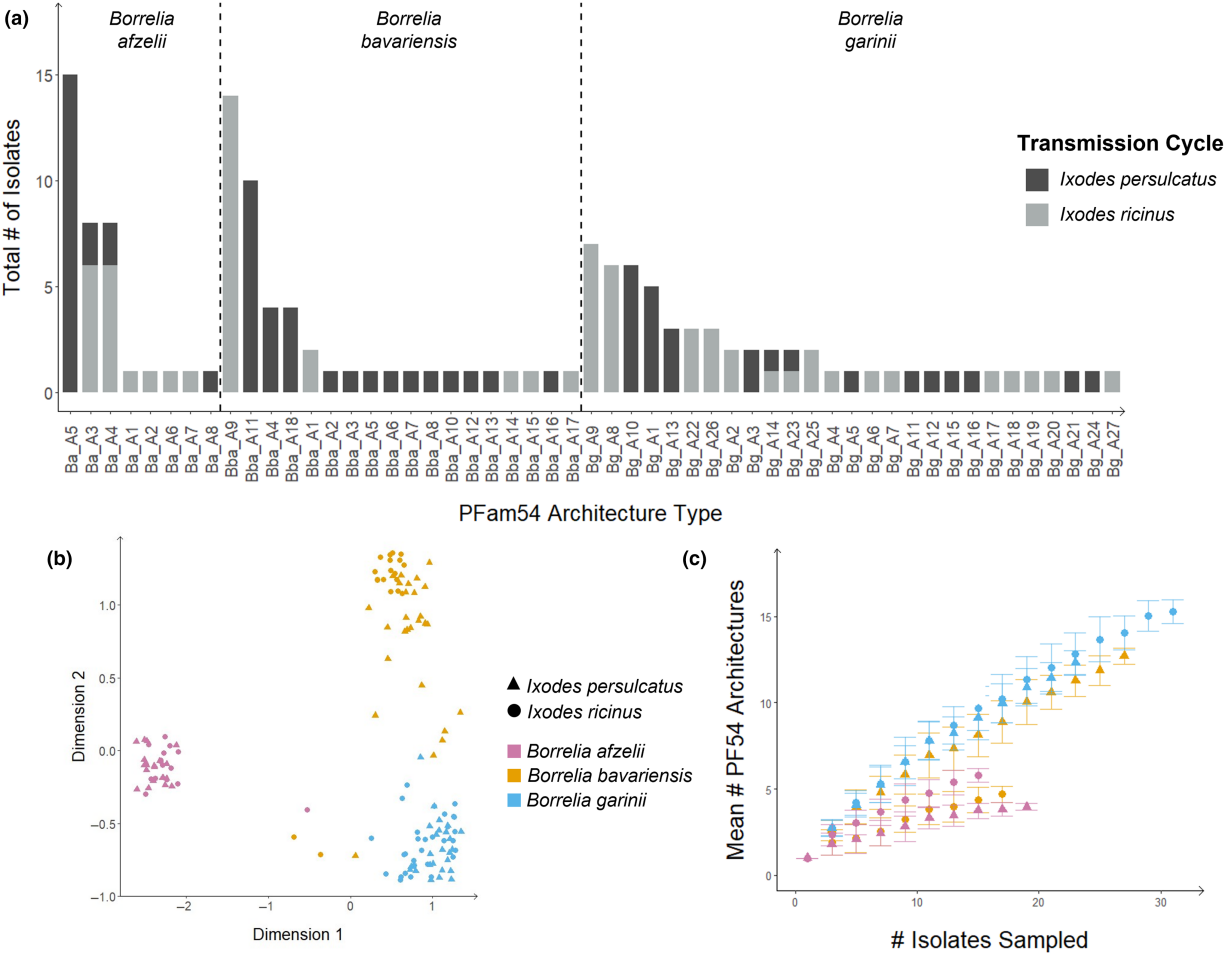
Diversity and prevalence of PFam54 architecture types identified in the 141 Eurasian isolates of *B. afzelii*, *B. bavariensis*, and *B. garinii* analyzed in this study. (a) Frequency of different architecture types present in the studied isolates (*n* = 141). Architecture types are separated by species (*B. afzelii*, *B. bavarinesis*, *B. garinii*). Colors correspond to tick transmission cycle (dark gray, *I. persulcatus*; light gray, *I. ricinus*). For information on the architecture of individual isolates see Table S3. (b) MDS clustering based on presence/absence matrix of orthology groups (OG) in each isolate. Here each point corresponds to a single *Borrelia* isolate. (c) Saturation curve analysis produced per species per transmission. In panels B and C, colors correspond to *Borrelia* species (purple, *B. afzelii*; orange, *B. bavariensis*; blue, *B. garinii*) and shapes correspond to the hypothesis tick transmission cycle based on the geographic origin of the isolates (*I. persulcatus* ▲, *I. ricinus* ●).

In total, 23 unique OGs could be described with 19 OGs being present in the 141 Eurasian *B. afzelii*, *B. bavariensis*, and *B. garinii* isolates studied (Table S1). In comparison to the three *Borrelia* species analyzed, *B. burgdorferi* sensu stricto (s.s.) strain B31, used as a reference, showed four unique OGs including OG14, OG15 (including *bba68* or synonymous *cspA*), OG19, and OG23 (Table S1). Five novel PFam54 paralogs were identified only in Asian isolates of all species (*pko2065b*, *bga67b*, *bga68b*, *bga71b*, and *zqa66b*), most of which belong to Clade IV (*n* = 4). Certain reference genes (*pko2069*, *pko2070*, *zqa73*) based on (Wywial et al., 2009) could not be detected in any of the 141 *Bb*sl isolates analyzed (Table S2). Both *B. afzelii* and *B. bavariensis* displayed one major architecture type (Ba_A5, *B. afzelii*; Bba_A11, *B. bavariensis*) (Figure 1a) while *B. garinii* displayed multiple architecture types at similar frequencies (Figure 1a). In general, we observed a high number of variable PFam54 gene array architecture types with 8, 18, and 27 different architectures found in *B. afzelii*, *B. bavariensis*, and *B. garinii*, respectively (Figure 1a, Figures S1–S3, Table S3). Within these, Clade I, II, III, and V genes (Wywial et al., 2009) were generally present in all *Borrelia* isolates with the majority of variability arising due to differences in gene length (e.g., *zqa66* in *B. garinii*, Figure S3) or absence/presence of Clade IV genes (Figures S1–S3, Table S2). Only *pko2060* and *zqa65* were found in all *B. afzelii* and *B. garinii* isolates, respectively, with all other identified paralogs being absent from at least one isolate (Table S2). MDS analysis based on the binary string of OG presence/absence showed that *Borrelia* species generally form independent groups with only a few samples not displaying clear clustering based on species identity (Figure 1b). In an effort to determine if our samples represented all possible PFam54 architecture types, we performed a saturation curve analysis. Only Asian *B. bavariensis* and *B. garinii* appeared to not reach an asymptote in this analysis suggesting there could be further architecture types present in both populations (Figure 1c, see Table S3 for additional information on individual architecture types per isolate).

**FIGURE 2:**
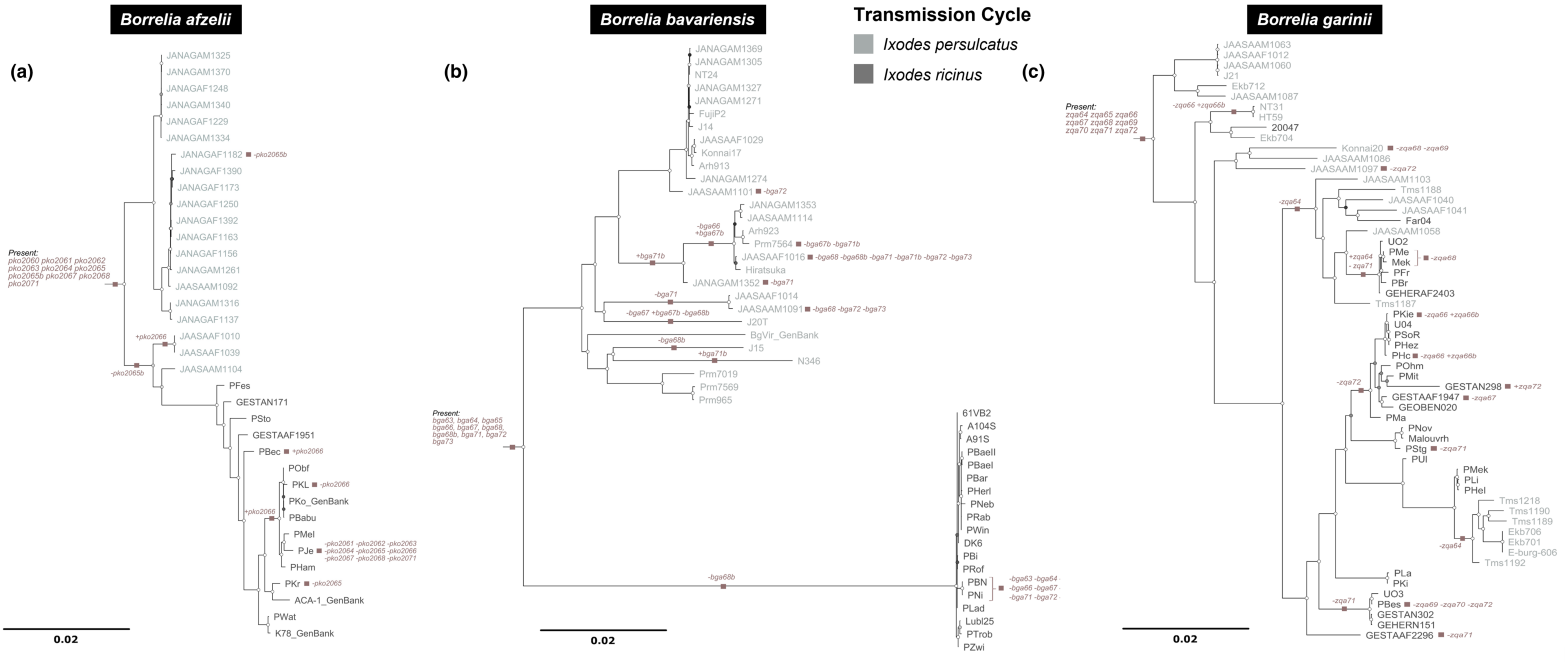
Gene gain and loss events of PFam54 paralogs based on the principle of maximum parsimony mapped onto the phylogenetic tree reconstructed based on full lp54 sequences corrected for recombination based on the four‐gamete condition test described in (Gatzmann et al., 2015; Rollins et al., 2023) and with the PFam54 gene array removed. Phylogenetic reconstruction was performed in MrBayes v.3.2.6 (Huelsenbeck & Ronquist, 2001; Ronquist et al., 2012) with ploidy set to haploid and a GTR (Tavaré, 1986) substitution model with inverse gamma‐distributed rate variation. Three independent runs were launched and ran for 10 million generations at which point convergence of parameters was checked with Tracer v.1.7.1 (Rambaut et al., 2018). Consensus trees were built using the *sumt* command from MrBayes using a respective burn‐in of 25%. Convergence to a single topology in all three independent runs was checked manually in FigTree v.1.4.4 (http://tree.bio.ed.ac.uk/software/figtree/). Colors correspond to the hypothesis tick transmission cycle based on the geographic origin of the isolates (*I. persulcatus* or *I. ricinus*).

Most paralogs described (74%) are hypothesized to be present at the base of the lp54 phylogenies of the respective species (i.e., present for the entire evolutionary history of each *Borrelia* species studied) including two novel PFam54 paralogs, *bga68b* in *B. bavariensis* and *pko2065b* in *B. afzelii* (Figure 2). Variation in which paralogs were found per isolate appears to have arisen mostly through individual gene gain (*n* = 12) or loss (*n* = 56) events with losses being more common (Figure 2). *Borrelia afzelii* displayed the lowest number of gene gains or losses (*n* = 16) followed by *B. garinii* (*n* = 23) with *B. bavariensis* displaying the highest (*n* = 29).

**FIGURE 3:**
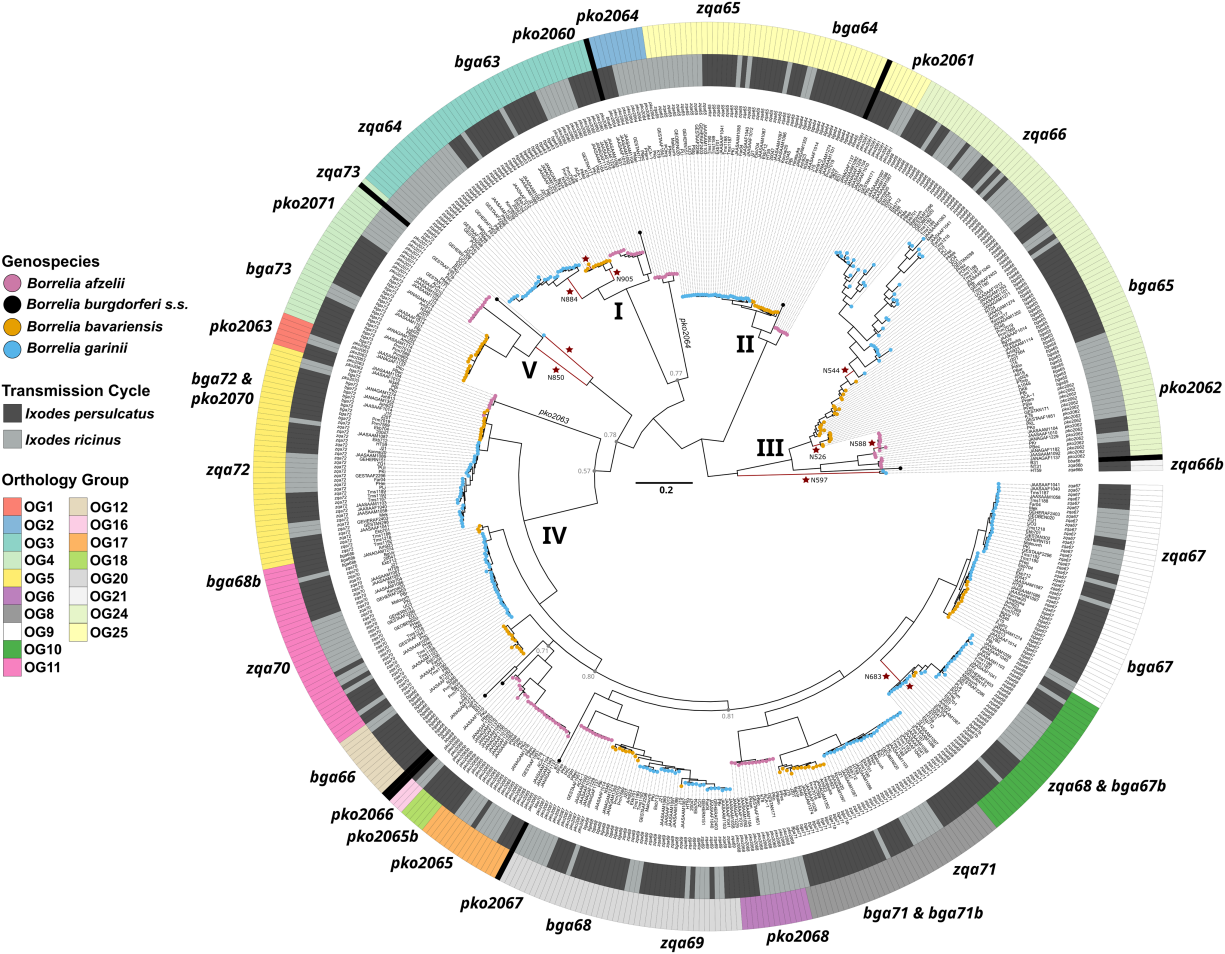
Phylogenetic tree of all, unique PFam54 paralogs identified in our analysis. In total, 1302 paralogs were identified which represented 524 unique sequences. Phylogenetic reconstruction was run in MrBayes v.3.2.6 (Huelsenbeck & Ronquist, 2001; Ronquist et al., 2012) with ploidy set to haploid and a codon substitution model with inverse gamma‐distributed rate variation, the universal genetic code, and assuming equal selection (ω) (Goldman & Yang, 1994; Muse & Gaut, 1994). Three independent runs were launched and ran for 50 million generations at which point convergence of parameters was checked with Tracer v.1.7.1 (Rambaut et al., 2018). Consensus trees were built using the *sumt* command from MrBayes using a respective burn‐in of 25%. Convergence to a single topology in all three independent runs was checked manually in FigTree v.1.4.4 (http://tree.bio.ed.ac.uk/software/figtree/). All internal nodes that had probabilities lower than 0.95 are shown in light gray. Orthology groups (OGs), based on the monophyletic clustering of an individual gene copy within the tree either for a single or multiple species, are shown in the outer ring. Hypothesized tick transmission cycle based on the geographic origin of the isolate is shown as either dark gray (*I. persulcatus*) or light gray (*I. ricinus*) in the inner ring. Individual paralog placements in the phylogeny are marked outside of these rings. *Borrelia s*pecies are denoted by tip end color: purple (*B. afzelii*), orange (*B. bavariensis*), blue (*B. garinii*). Roman numerals (I–V) denote the PFam54 Clades as described in (Wywial et al., 2009). Red stars denote branches that were found to show significant instances of diversifying selection as determined by aBSREL v2.2 (Smith et al., 2015) from the HyPhy package (https://www.hyphy.org/) using the universal genetic code and not allowing for multiple hits.

All five clades (I–V) originally described in (Wywial et al., 2009) were found in our dataset, although structuring of the clades deviated with Clade III being the most basal group (Figure 3) instead of Clade I as previously described (Wywial et al., 2009). The genes *pko2063* and *pko2064*, which were previously not assigned to a Clade, form sister clades to Clade I and IV, respectively, but with lower node probability (*pko2063*, *p* = 0.57; *pko2064*, *p* = 0.77) and remain unique genes of *B. afzelii* (Figure 3). In the phylogenetic reconstruction of the PFam54 gene family, most genes form monophyletic clades by *Borrelia* species within each OG (Figure 3), although some paralogs do not follow this pattern. For example, *zqa66* (*B. garinii*) and *bga65* (*B. bavariensis*) form a monophyletic clade (Figure 3) with all *zqa66* sequences forming a monophyletic clade within the larger *zqa66/bga65* clade (Figure 3). A similar pattern can be seen for other *B. bavariensis* and *B. garinii* paralogs (*bga68* and *zqa69*; *bga67* and *zqa67*; *bga64* and *zqa65*) where the genes do not form monophyletic clades purely based on species (Figure 3). Additionally, *pko2071* of *B. afzelii* and *bga73* of *B. bavariensis* form a monophyletic clade with the *B. garinii* paralog (*zqa73*) as a sister clade to *pko2071*/*bga73* (Figure 3). Novel PFam54 paralogs clustered throughout the phylogeny with *bga67b* and *bga68b* of *B. bavariensis* being paralogs of *B. garinii* genes *zqa68* and *zqa70*, respectively (Figure 3). *Borrelia bavariensis* gene *bga71b* clusters within *bga71* but *bga71b* is paraphyletic (Figure 3). *Borrelia afzelii* gene *pko2065b* forms a sister clade to *pko2065* with *pko2066* being basal to both genes (Figure 3). Finally, *zqa66b* forms a single monophyletic clade which is a sister clade to the OG24 group (Clade III) containing *pko2062*, *bga65*, and *zqa66* (Figure 3).

Some proteins, predominantly encoded by Clade IV PFam54 genes, are known to play a role in immune evasion with influences to host and, potentially, vector adaptation (Gilmore et al., 2010; Hart et al., 2018; Patton et al., 2011, 2013). Host or vector adaptation could leave signatures of diversifying selection in genes related to this phenotype, such as genes encoding the PFam54. This allows us to test whether PFam54 paralogs of *B. afzelii*, *B. bavariensis*, and *B. garinii* are also implicated for host and/or vector adaptation by testing branches in the phylogeny which separate out clades that differ in which vector or host they are hypothesized to use. Of the 44 branches tested, 11 branches showed significant evidence for diversifying selection (Table S4). These were found in five OGs including OG3 (Clade 1), OG24 (Clade III), OG10 (Clade IV), OG4 (Clade V), and OG21 (Figure 3). Of these, four separated out clades of isolates are hypothesized to utilize either *I. ricinus* (Europe) or *I. persulcatus* (Asia) as vectors (N588, *B. afzelii* on *pko2062*; N526, *B. bavariensis* on *bga65*; terminal branch leading to *B. bavariensis* isolate PBi on *bga63* and *B. garinii* isolate 20,047 on *zqa68*) (Figure 3; indicated by red stars). The other seven branches separate clades of isolates which differ in their proposed, reservoir hosts (rodent or bird) (Figure 3). Specifically, OG3 (Clade I) and OG24 (Clade III) where both branches leading either to rodent‐associated *B. afzelii* (N850) or *B. bavariensis* (N905) as well as bird‐associated *B. garinii* (*zqa63* terminal branch for ZQ1, N884) displayed significant instances of diversifying selection. Genes with known functionality to human or host adaptation predominantly did not show signatures of diversifying selection (*pko2068*, *B. afzelii*; *bga66* & *bga71*, *B. bavariensis*). Only the monophyletic clade containing *zqa68* and the novel *B. bavariensis* gene *bga67b* showed evidence for diversifying selection but *bga67b* alone did not (Figure 3, Table S4).

## 4 DISCUSSION

Utilizing recently published whole genome sequences (*n* = 136) (Rollins et al., 2023) and GenBank reference genomes (*n* = 5) obtained from 141 Eurasian *B. afzelii*, *B. bavariensis*, and *B. garinii* isolates, we aimed to quantify the genetic variation along the PFam54 gene array and to analyze how PFam54 genes have evolved in these three *Borrelia* species known to differ within their host and vector adaptation (Comstedt et al., 2011; Kurtenbach et al., 2006; Margos et al., 2019). Our analyses highlighted the PFam54 gene array is more variable than previously expected, with novel paralogs found in all three *Borrelia* species studied. Additionally, genes displaying signatures of diversifying selection potentially in association with hypothesized vector and/or host adaption lie outside of Clade IV which has received the majority of interest in relation to *Borrelia*‐host infection mechanisms.

The isolates studied displayed a high number of different PFam54 architecture types which has also been partially observed for *B. burgdorferi* s.s., although based on analysis of five isolates (Wywial et al., 2009) with overall synteny of Clade IV genes being shown as well for further isolates (Hart et al., 2021). Architecture types do appear to be geographically restricted suggesting that they potentially have evolved over time due to the selection pressures associated with various biotic and abiotic conditions arising from different tick‐host transmission cycles. Of particular interest is that *B. garinii* appears to maintain many equally frequent PFam54 architecture types in contrast to *B. afzelii* and *B. bavariensis* (Figure 1a, Figure S3). This species‐specific pattern supports the findings of adaptation of certain *Borrelia* species to specific vertebrate hosts (i.e., individual bird species) (Lin, Diuk‐Wasser, et al., 2020; Lin, Frye, et al., 2020). This pattern is mirrored in the full genome where the plasmid content of *B. garinii* isolates tends to be smaller than in other *Borrelia* species (Rollins et al., 2023). Even so, these smaller genomes do not correspond to an overall reduction in plasmid diversity (Rollins et al., 2023). These findings could suggest a trend toward smaller but, potentially, specialized genomes. Genome reduction is commonly found in pathogenic microorganisms and may be due to adaptation to a parasitic lifestyle (Moran, 2002). If true, one could hypothesize that the variability in architecture types within *B. garinii* isolates could correspond to the capacity to infect specific bird species. *Borrelia garinii* selectively binds complement regulator Factor H of avian origin to protect itself from complement‐mediated killing and, thus facilitate infection of avian hosts (Hart et al., 2018, 2021; Sürth et al., 2021). Differences in susceptibility to complement in vitro therefore could be one of the possibilities to determine infection capacity of *B. garinii* isolates. Recent data has demonstrated variation in the susceptibility of *B. garinii* to avian complement from different terrestrial European bird species (Sürth et al., 2021), suggesting that host adaptation toward specific bird species could exist.

Our data revealed, regardless of the genetic variability, that the PFam54 gene array within the isolates studied has remained relatively stable over time as most paralogs (74% of all) are predicted to be present at the base of the lp54 phylogenies (Figure 2). Even so, only two PFam54 paralogs, *pko2060* and *zqa65*, have been detected in all *B. afzelii* or *B. garinii* isolates, respectively, which could suggest one possibility that some genes are dispensable for completing the spirochetes tick‐host transmission cycle in the studied species. This includes the loss of genes encoding for proteins known to interact with the complement system of vertebrates, namely BGA66 and BGA71 of *B. bavariensis*, PKO2068 of *B. afzelii*, and ZQA68 of *B. garinii* (Hammerschmidt et al., 2016; Hart et al., 2021; Kraiczy, 2016). This is supported by recent work which showed that the loss of the whole PFam54 gene array in two *B. bavariensis* isolates (PBN, PNi) impacted susceptibility to human complement but did not influence infection of mice further highlighting the probable, functional redundancy in the overall *Borrelia* genome and especially in relation to the studied species (Rollins et al., 2022).

We identified a number of, so far, undescribed PFam54 paralogs including *pko2065b* (*B. afzelii*), *bga67b* (*B. bavariensis*), *bga68b* (*B. bavariensis*), *bga71b* (*B. bavariensis*), and *zqa66b* (*B. garinii*). As *pko2065b* and *bga68b* are suggested to be present at the base of the species‐specific lp54 phylogenies, it could be hypothesized that both genes have been maintained over the evolutionary history of *B. afzelii* and *B. bavariensis*, suggesting these genes could have a yet unknown function with importance to these species completing their lifecycle. For this, *bga68b*, and additionally *bga67b*, are paralogs, respectively, of *zqa68* and *zqa70* from *B. garinii*. Concerning the history of *bga67b*, this gene could have arisen through a recombination event between *B. bavariensis* and *B. garinii*, as suggested previously for genes located on the chromosome (Rollins et al., 2023). Of note, the ortholog of BGA67b in *B. garinii*, ZQA68, displays selective binding properties to the complement regulator Factor H of avian origin and thus protects spirochetes from complement‐mediated killing by avian innate immunity (Hart et al., 2018, 2021). Polymorphisms in complement‐interacting proteins of *B. burgdorferi* s.s. are known to influence potential host adaptation through complement binding (Hart et al., 2018, 2021; Lin, Diuk‐Wasser, et al., 2020; Lin, Frye, et al., 2020; Marcinkiewicz et al., 2018). Even though multiple single nucleotide polymorphisms are present when comparing the *zqa68* and *bga67b* sequences, it is tempting to speculate that this paralog could enable Asian *B. bavariensis* isolates to circulate in birds as previously suggested in contrast to European *B. bavariensis* which is most likely a mammalian‐associated *Borrelia* species (Ishiguro et al., 2005; Margos et al., 2019; Munro et al., 2017; Rollins et al., 2023). However, further in vitro studies are required to understand the role(s) of these novel paralogs in host adaptation and immune evasion. We also observed the complete absence of specific, reference genes from all analyzed isolates (*pko2069*, *pko2070*, *zqa73*) (Table S2). Noteworthy, our analysis was based on short‐read sequencing technologies which have been shown in recent years to not capture the entirety of the complex *Borrelia* genome, with the use of long‐read technologies (e.g., PacBio) providing much better, but also not perfect, resolution (Combs et al., 2022; Hepner et al., 2023; Margos et al., 2017). This could explain the absence of some genes in our analysis and also the presence of many, singular architecture types potentially due to lower resolution in specific samples. Nevertheless, short‐reads have been shown to adequately reconstruct the lp54 where the PFam54 gene array is located (Margos et al., 2017) and previous work has shown that short‐read data was able to reconstruct the full absence of the PFam54 gene array in *B. bavariensis* isolates PBN and PNi with confirmation through long‐read sequencing (Rollins et al., 2022). Taken together, this suggests short‐read sequencing should be adequate to reconstruct the PFam54 gene array but future analysis should include long‐read sequencing data to determine any bias that could arise due to sequencing methodology.

The majority of branches that have experienced diversifying selection potentially in relation to host and/or vector adaptation (i.e., along branches separating clades hypothesized to utilize different vertebrate hosts or tick vectors) belonged to non‐Clade IV PFam54 genes that have not been directly linked to host and vector adaptation so far (Caimano et al., 2019; Hammerschmidt et al., 2014; Hart et al., 2021; Iyer et al., 2015; Wywial et al., 2009). Indeed, Clade IV paralogs encoding for proteins capable of binding vertebrate complement proteins/regulators only contained two branches displaying instances of diversifying selection both in the *zqa68*/*bga67b* clade (Figure 3). Genes belonging to Clades I and III (*bga63*, *bga65*, *pko2062*) displayed the majority of branches separating out isolates which are hypothesized to be transmitted by different ticks, potentially suggesting diversifying selection in relation to this phenotype. Previous work revealed that PFam54 Clade I and III paralogs *bba64* and *bba66* from *B. burgdorferi* s.s. are variably regulated during tick infection (Iyer et al., 2015). Additional studies have shown that some PFam54 members grouped in Clade I and III are important for tick transmission of *B. burgdorferi* s.s. to the mammalian host (Gilmore et al., 2010; Hart et al., 2018; Patton et al., 2011, 2013), potentially highlighting the importance of these paralogous proteins in host‐pathogen interactions. Clades I, III, and V contained branches also displaying instances of diversifying selection potentially linked to host adaptation (i.e., along branches separating clades with differences in the proposed reservoir host). Clades I, III, and V paralogs of *B. burgdorferi* s.s. have been shown to be upregulated during infection of tick‐infected mice (Caimano et al., 2019; Gilmore et al., 2008), but loss of these genes did not significantly impede host infection, although some variation in tissue tropism was observed (Caimano et al., 2019; Patton et al., 2011, 2013). These results highlight that PFam54 genes outside of Clade IV are most likely important for *Borrelia* transmission from the vector to the host but additional investigations are required to assess their role in host and/or vector adaptation within Eurasian‐distributed *Borrelia* species. Further studies are required to confirm the results of this correlative analysis and to assess the function of these particular paralogs regarding their complement‐inhibitory capacity.

Taken together, our results show that the PFam54 gene array of the three main causative agents of LB across Eurasia (*B. afzelii*, *B. bavariensis*, *B. garinii*) is highly variable in genetic architecture including gene content. Moreover, the signatures of diversifying selection identified emphasize a potential role of paralogs belonging to Clades I, III, and V in host and vector adaptation and, thus, in functionality toward the natural transmission cycles of the three *Borrelia* species investigated herein. Utilizing genomic data, we were able to elucidate the evolution of an important gene family and were able to generate testable hypotheses regarding which genes should be studied in relation to host and vector adaptation in the future. Taken together, our comparative analyses highlight the importance of investigating individual diversity in *Borrelia* species from a population genetics perspective. Analyses such as these can increase our understanding and guide future projects into *Borrelia* pathogenesis, with the overall goal to determine how vector‐borne pathogens evolve leading to the emergence of wildlife and human disease.

## AUTHOR CONTRIBUTIONS

**Janna Wülbern:** Data curation (equal); formal analysis (supporting); investigation (equal); writing – original draft (supporting). **Laura Windorfer:** Formal analysis (equal); investigation (supporting); methodology (equal). **Kozue Sato:** Data curation (supporting); investigation (equal); writing – original draft (supporting). **Minoru Nakao:** Data curation (supporting); investigation (supporting). **Sabrina Hepner:** Investigation (supporting); writing – original draft (supporting). **Gabriele Margos:** Conceptualization (supporting); funding acquisition (supporting); project administration (supporting); supervision (supporting); writing – original draft (equal). **Volker Fingerle:** Conceptualization (supporting); funding acquisition (supporting); project administration (supporting); resources (equal); supervision (supporting); writing – original draft (equal). **Hiroki Kawabata:** Conceptualization (supporting); data curation (supporting); funding acquisition (supporting); project administration (supporting); writing – original draft (supporting). **Noémie S. Becker:** Conceptualization (lead); formal analysis (supporting); funding acquisition (lead); project administration (equal); supervision (equal); writing – original draft (equal). **Peter Kraiczy:** Conceptualization (equal); investigation (supporting); project administration (supporting); supervision (equal); writing – original draft (equal); writing – review and editing (equal). **Robert E. Rollins:** Conceptualization (equal); data curation (lead); formal analysis (lead); investigation (lead); project administration (equal); supervision (equal); visualization (lead); writing – original draft (lead); writing – review and editing (lead).

## FUNDING INFORMATION

The project was funded through the German Research Foundation (DFG Grant No. BE 5791/2‐1) (NSB, RER). The National Reference Centre for *Borrelia* was funded by the Robert‐Koch‐Institut, Berlin (VF, GM). Part of this work was supported by the LOEWE Center DRUID (Novel Drug Targets against Poverty‐Related and Neglected Tropical Infectious Diseases), projects C3 (PK).

## CONFLICT OF INTEREST STATEMENT

The authors have no conflicts of interest to state.

## Supporting information


Appendix S1.

## Data Availability

All SRA files have been uploaded to GenBank under the BioProject numbers PRJNA327303, PRJNA449844, and PRJNA722378. All assembled lp54 sequences, finalized PFam54 gene lists and sequences, finalized trees (lp54 and PFam54), all MrBayes output files and alignments used, and HyPhY output files have been uploaded to a Mendeley Data repository and can be accessed at DOI: 10.17632/xy8wt4ty8f.1.
