# Supplementary material for: Unprecedented genetic variability of PFam54 paralogs among Eurasian Lyme borreliosis‐causing spirochetes
Source: Ecol Evol. 2024 May 21;14(5):e11397. doi: 10.1002/ece3.11397 (PMC11109050; doi:10.1002/ece3.11397)
Supplement: Supplementary file 1 — Appendix S1. [file ECE3-14-e11397-s001.docx]

**Unprecedented genetic variability of PFam54 paralogs among Eurasian Lyme borreliosis spirochetes**

**Ecology and Evolution**

Janna Wülbern, Laura Windorfer, Kozue Sato, Minoru Nakao, Sabrina Hepner, Gabriele Margos, Volker Fingerle, Hiroki Kawabata, Noémie S. Becker, Peter Kraiczy and

Robert E. Rollins

- **Online Supplementary Material —**
- **Supplementary Tables & Figures —**

**Table S1.** Orthology groups and assigned PFam54 paralogs based on phylogenetic reconstruction.

| **Orthology Group** | **Genes** |
| --- | --- |
| **OG1** | *pko2063* |
| **OG2** | *pko2064* |
| **OG3** | *pko2060, bga63, zqa64* |
| **OG4** | *pko2071, bga73, zqa73* |
| **OG5** | *bga72, zqa72, pko2070* |
| **OG6** | *pko2068* |
| **OG7** | *bga69, bga70* |
| **OG8** | *bga71, bga71b, zqa71* |
| **OG9** | *bga67, zqa67* |
| **OG10** | *bga67b, zqa68* |
| **OG11** | *bga68b, zqa70* |
| **OG12** | *bga66* |
| **OG14** | *bba69* |
| **OG15** | *bba68* |
| **OG16** | *pko2066* |
| **OG17** | *pko2065* |
| **OG18** | *pko2065b* |
| **OG19** | *bba70* |
| **OG20** | *pko2067, bga68, zqa69* |
| **OG21** | *zqa66b* |
| **OG23** | *bba66* |
| **OG24** | *pko2062, bga65, zqa66* |
| **OG25** | *pko2061, bga64, zqa65* |

**Table S2.** Proportion of isolates containing a given PFam54 paralog. Proportions either refer to all isolates, Asian isolates (only isolates from *I. persulcatus* transmission cycle), or European isolates (only isolates from *I. ricinus* transmission cycle).

| **Species** | **PFam54 Gene** | **All** | **Asia** | **Europe** |
| --- | --- | --- | --- | --- |
| ***Borrelia afzelii*** | *pko2060* | 1.00 | 1.00 | 1.00 |
|  | *pko2061* | 0.97 | 1.00 | 0.94 |
|  | *pko2062* | 0.97 | 1.00 | 0.94 |
|  | *pko2063* | 0.97 | 1.00 | 0.94 |
|  | *pko2064* | 0.97 | 1.00 | 0.94 |
|  | *pko2065* | 0.94 | 1.00 | 0.88 |
|  | *pko2065b* | 0.44 | 0.80 | 0.00 |
|  | *pko2066* | 0.22 | 0.10 | 0.38 |
|  | *pko2067* | 0.97 | 1.00 | 0.94 |
|  | *pko2068* | 0.97 | 1.00 | 0.94 |
|  | *pko2069* | 0.00 | 0.00 | 0.00 |
|  | *pko2070* | 0.00 | 0.00 | 0.00 |
|  | *pko2071* | 0.97 | 1.00 | 0.94 |
| ***Borrelia bavariensis*** | *bga63* | 0.96 | 1.00 | 0.89 |
|  | *bga64* | 0.96 | 1.00 | 0.89 |
|  | *bga65* | 0.96 | 1.00 | 0.89 |
|  | *bga66* | 0.83 | 0.79 | 0.89 |
|  | *bga67* | 0.91 | 0.92 | 0.89 |
|  | *bga67b* | 0.13 | 0.21 | 0.00 |
|  | *bga68* | 0.91 | 0.93 | 0.89 |
|  | *bga68b* | 0.49 | 0.82 | 0.00 |
|  | *bga71* | 0.87 | 0.86 | 0.89 |
|  | *bga71b* | 0.13 | 0.21 | 0.00 |
|  | *bga72* | 0.89 | 0.89 | 0.89 |
|  | *bga73* | 0.91 | 0.92 | 0.89 |
| ***Borrelia garinii*** | *zqa64* | 0.76 | 0.48 | 0.97 |
|  | *zqa65* | 1.00 | 1.00 | 1.00 |
|  | *zqa66* | 0.93 | 0.92 | 0.94 |
|  | *zqa66b* | 0.07 | 0.08 | 0.06 |
|  | *zqa67* | 0.98 | 1.00 | 0.97 |
|  | *zqa68* | 0.95 | 0.96 | 0.94 |
|  | *zqa69* | 0.97 | 0.96 | 0.97 |
|  | *zqa70* | 0.98 | 1.00 | 0.97 |
|  | *zqa71* | 0.79 | 1.00 | 0.64 |
|  | *zqa72* | 0.79 | 0.96 | 0.67 |
|  | *zqa73* | 0.00 | 0.00 | 0.00 |

**Table S3.** Individual architecture types and meta-data for each isolate described in the study. Here year refers to year of isolation, origin refers to transmission cycle (Europe = *I. ricinus*, Asia = *I. persulcatus*), and source refers to the source material used for isolation.

| **Isolate** | **Species** | **Year** | **Origin** | **Source** | **Architecture Type** |
| --- | --- | --- | --- | --- | --- |
| PJe | *Borrelia afzelii* | 1992 | Europe | Human | Ba_A1 |
| PKr | *Borrelia afzelii* | 1992 | Europe | Human | Ba_A2 |
| ACA-1 | *Borrelia afzelii* | - | Europe | Human | Ba_A3 |
| GESTAAF1951 | *Borrelia afzelii* | 2019 | Europe | Tick | Ba_A3 |
| GESTAN171 | *Borrelia afzelii* | 2018 | Europe | Tick | Ba_A3 |
| JAASAAM1104 | *Borrelia afzelii* | 2019 | Asia | Tick | Ba_A3 |
| JANAGAF1182 | *Borrelia afzelii* | 2019 | Asia | Tick | Ba_A3 |
| K78 | *Borrelia afzelii* | - | Europe | Human | Ba_A3 |
| PFes | *Borrelia afzelii* | 1988 | Europe | Human | Ba_A3 |
| PWat | *Borrelia afzelii* | 1994 | Europe | Human | Ba_A3 |
| JAASAAF1010 | *Borrelia afzelii* | 2019 | Asia | Tick | Ba_A4 |
| JAASAAF1039 | *Borrelia afzelii* | 2019 | Asia | Tick | Ba_A4 |
| PBabu | *Borrelia afzelii* | 2001 | Europe | Human | Ba_A4 |
| PBec | *Borrelia afzelii* | 1988 | Europe | Human | Ba_A4 |
| PHam | *Borrelia afzelii* | 1987 | Europe | Human | Ba_A4 |
| PKo | *Borrelia afzelii* | 1984 | Europe | Human | Ba_A4 |
| PMeI | *Borrelia afzelii* | 1990 | Europe | Human | Ba_A4 |
| PObf | *Borrelia afzelii* | 1998 | Europe | Human | Ba_A4 |
| JAASAAF1092 | *Borrelia afzelii* | 2019 | Asia | Tick | Ba_A5 |
| JANAGAF1156 | *Borrelia afzelii* | 2019 | Asia | Tick | Ba_A5 |
| JANAGAF1163 | *Borrelia afzelii* | 2019 | Asia | Tick | Ba_A5 |
| JANAGAF1173 | *Borrelia afzelii* | 2019 | Asia | Tick | Ba_A5 |
| JANAGAF1229 | *Borrelia afzelii* | 2019 | Asia | Tick | Ba_A5 |
| JANAGAF1248 | *Borrelia afzelii* | 2019 | Asia | Tick | Ba_A5 |
| JANAGAF1250 | *Borrelia afzelii* | 2019 | Asia | Tick | Ba_A5 |
| JANAGAF1390 | *Borrelia afzelii* | 2019 | Asia | Tick | Ba_A5 |
| JANAGAF1392 | *Borrelia afzelii* | 2019 | Asia | Tick | Ba_A5 |
| JANAGAM1261 | *Borrelia afzelii* | 2019 | Asia | Tick | Ba_A5 |
| JANAGAM1316 | *Borrelia afzelii* | 2019 | Asia | Tick | Ba_A5 |
| JANAGAM1325 | *Borrelia afzelii* | 2019 | Asia | Tick | Ba_A5 |
| JANAGAM1334 | *Borrelia afzelii* | 2019 | Asia | Tick | Ba_A5 |
| JANAGAM1340 | *Borrelia afzelii* | 2019 | Asia | Tick | Ba_A5 |
| JANAGAM1370 | *Borrelia afzelii* | 2019 | Asia | Tick | Ba_A5 |
| PSto | *Borrelia afzelii* | 1986 | Europe | Human | Ba_A6 |
| PKL | *Borrelia afzelii* | 1993 | Europe | Human | Ba_A7 |
| JANAGAF1137 | *Borrelia afzelii* | 2019 | Asia | Tick | Ba_A8 |
| PBN | *Borrelia bavariensis* | 1999 | Europe | Human | Bba_A1 |
| PNi | *Borrelia bavariensis* | 2000 | Europe | Human | Bba_A1 |
| Prm7564 | *Borrelia bavariensis* | 2011 | Asia | Tick | Bba_A2 |
| JAASAAF1016 | *Borrelia bavariensis* | 2019 | Asia | Tick | Bba_A3 |
| Arh923 | *Borrelia bavariensis* | 2012 | Asia | Tick | Bba_A4 |
| Hiratsuka | *Borrelia bavariensis* | 2008 | Asia | Human | Bba_A4 |
| JAASAAM1114 | *Borrelia bavariensis* | 2019 | Asia | Tick | Bba_A4 |
| JANAGAM1353 | *Borrelia bavariensis* | 2019 | Asia | Tick | Bba_A4 |
| JAASAAF1029 | *Borrelia bavariensis* | 2019 | Asia | Tick | Bba_A5 |
| J20T | *Borrelia bavariensis* | 1996 | Asia | Human | Bba_A6 |
| JAASAAM1091 | *Borrelia bavariensis* | 2019 | Asia | Tick | Bba_A7 |
| JAASAAF1014 | *Borrelia bavariensis* | 2019 | Asia | Tick | Bba_A8 |
| 61VB2 | *Borrelia bavariensis* | - | Europe | Human | Bba_A9 |
| A104S | *Borrelia bavariensis* | 1996 | Europe | Human | Bba_A9 |
| A91S | *Borrelia bavariensis* | 1996 | Europe | Human | Bba_A9 |
| Lubl25 | *Borrelia bavariensis* | 1995 | Europe | Human | Bba_A9 |
| PBaeI | *Borrelia bavariensis* | 1990 | Europe | Human | Bba_A9 |
| PBaeII | *Borrelia bavariensis* | 1990 | Europe | Human | Bba_A9 |
| PBar | *Borrelia bavariensis* | 1988 | Europe | Human | Bba_A9 |
| PBi | *Borrelia bavariensis* | <1993 | Europe | Human | Bba_A9 |
| PLad | *Borrelia bavariensis* | 2000 | Europe | Human | Bba_A9 |
| PNeb | *Borrelia bavariensis* | 1988 | Europe | Human | Bba_A9 |
| PRof | *Borrelia bavariensis* | 1989 | Europe | Human | Bba_A9 |
| PTrob | *Borrelia bavariensis* | 1988 | Europe | Human | Bba_A9 |
| PWin | *Borrelia bavariensis* | 1987 | Europe | Human | Bba_A9 |
| PZwi | *Borrelia bavariensis* | 1994 | Europe | Human | Bba_A9 |
| JAASAAM1101 | *Borrelia bavariensis* | 2019 | Asia | Tick | Bba_A10 |
| Arh913 | *Borrelia bavariensis* | 2012 | Asia | Tick | Bba_A11 |
| FujiP2 | *Borrelia bavariensis* | - | Asia | Tick | Bba_A11 |
| J14 | *Borrelia bavariensis* | 1995 | Asia | Tick | Bba_A11 |
| JANAGAM1271 | *Borrelia bavariensis* | 2019 | Asia | Tick | Bba_A11 |
| JANAGAM1274 | *Borrelia bavariensis* | 2019 | Asia | Tick | Bba_A11 |
| JANAGAM1305 | *Borrelia bavariensis* | 2019 | Asia | Tick | Bba_A11 |
| JANAGAM1327 | *Borrelia bavariensis* | 2019 | Asia | Tick | Bba_A11 |
| JANAGAM1369 | *Borrelia bavariensis* | 2019 | Asia | Tick | Bba_A11 |
| Konnai17 | *Borrelia bavariensis* | 2011 | Asia | Tick | Bba_A11 |
| NT24 | *Borrelia bavariensis* | - | Asia | Tick | Bba_A11 |
| JANAGAM1352 | *Borrelia bavariensis* | 2019 | Asia | Tick | Bba_A12 |
| N346 | *Borrelia bavariensis* | - | Asia | Tick | Bba_A13 |
| PRab | *Borrelia bavariensis* | 1994 | Europe | Human | Bba_A14 |
| PHerl | *Borrelia bavariensis* | 1989 | Europe | Human | Bba_A15 |
| J15 | *Borrelia bavariensis* | 1995 | Asia | Human | Bba_A16 |
| DK6 | *Borrelia bavariensis* | 1990 | Europe | Human | Bba_A17 |
| BgVir | *Borrelia bavariensis* | - | Asia | Human | Bba_A18 |
| Prm7019 | *Borrelia bavariensis* | 2012 | Asia | Tick | Bba_A18 |
| Prm7569 | *Borrelia bavariensis* | 2011 | Asia | Tick | Bba_A18 |
| Prm965 | *Borrelia bavariensis* | 2013 | Asia | Tick | Bba_A18 |
| JAASAAF1040 | *Borrelia garinii* | 2019 | Asia | Tick | Bg_A1 |
| JAASAAM1058 | *Borrelia garinii* | 2019 | Asia | Tick | Bg_A1 |
| JAASAAM1103 | *Borrelia garinii* | 2019 | Asia | Tick | Bg_A1 |
| Tms1187 | *Borrelia garinii* | 2013 | Asia | Tick | Bg_A1 |
| Tms1188 | *Borrelia garinii* | 2013 | Asia | Tick | Bg_A1 |
| PHc | *Borrelia garinii* | 1996 | Europe | Human | Bg_A2 |
| PKie | *Borrelia garinii* | 1993 | Europe | Human | Bg_A2 |
| HT59 | *Borrelia garinii* | - | Asia | Tick | Bg_A3 |
| NT31 | *Borrelia garinii* | - | Asia | Tick | Bg_A3 |
| GESTAAF1947 | *Borrelia garinii* | 2019 | Europe | Tick | Bg_A4 |
| Konnai20 | *Borrelia garinii* | - | Asia | Tick | Bg_A5 |
| PMe | *Borrelia garinii* | 1988 | Europe | Human | Bg_A6 |
| PBes | *Borrelia garinii* | 1989 | Europe | Human | Bg_A7 |
| GEHERAF2403 | *Borrelia garinii* | 2019 | Europe | Tick | Bg_A8 |
| GEHERN151 | *Borrelia garinii* | 2018 | Europe | Tick | Bg_A8 |
| GESTAAF2296 | *Borrelia garinii* | 2019 | Europe | Tick | Bg_A8 |
| GESTAN302 | *Borrelia garinii* | 2018 | Europe | Tick | Bg_A8 |
| PStg | *Borrelia garinii* | 1996 | Europe | Human | Bg_A8 |
| UO2 | *Borrelia garinii* | - | Europe | - | Bg_A8 |
| GEOBEN020 | *Borrelia garinii* | 2018 | Europe | Tick | Bg_A9 |
| PHez | *Borrelia garinii* | 1994 | Europe | Human | Bg_A9 |
| PMa | *Borrelia garinii* | 1989 | Europe | Human | Bg_A9 |
| PMit | *Borrelia garinii* | 1997 | Europe | Human | Bg_A9 |
| POhm | *Borrelia garinii* | 1991 | Europe | Human | Bg_A9 |
| PSoR | *Borrelia garinii* | 1989 | Europe | Human | Bg_A9 |
| UO4 | *Borrelia garinii* | - | Europe | - | Bg_A9 |
| Ekb704 | *Borrelia garinii* | 2011 | Asia | Tick | Bg_A10 |
| Ekb712 | *Borrelia garinii* | 2011 | Asia | Tick | Bg_A10 |
| J21 | *Borrelia garinii* | 1996 | Asia | Tick | Bg_A10 |
| JAASAAF1012 | *Borrelia garinii* | 2019 | Asia | Tick | Bg_A10 |
| JAASAAM1060 | *Borrelia garinii* | 2019 | Asia | Tick | Bg_A10 |
| JAASAAM1086 | *Borrelia garinii* | 2019 | Asia | Tick | Bg_A10 |
| Tms1218 | *Borrelia garinii* | 2013 | Asia | Tick | Bg_A11 |
| Tms1190 | *Borrelia garinii* | 2013 | Asia | Tick | Bg_A12 |
| E-burg-606 | *Borrelia garinii* | 2019 | Asia | Tick | Bg_A13 |
| Ekb701 | *Borrelia garinii* | 2011 | Asia | Tick | Bg_A13 |
| Ekb706 | *Borrelia garinii* | 2011 | Asia | Tick | Bg_A13 |
| Far04 | *Borrelia garinii* | <1999 | Europe | Puffin | Bg_A14 |
| JAASAAF1041 | *Borrelia garinii* | 2019 | Asia | Tick | Bg_A14 |
| Tms1189 | *Borrelia garinii* | 2013 | Asia | Tick | Bg_A15 |
| Tms1192 | *Borrelia garinii* | 2013 | Asia | Tick | Bg_A16 |
| Mek | *Borrelia garinii* | 1992 | Europe | Human | Bg_A17 |
| PBr | *Borrelia garinii* | 1985 | Europe | Human | Bg_A18 |
| UO3 | *Borrelia garinii* | - | Europe | - | Bg_A19 |
| PFr | *Borrelia garinii* | 1995 | Europe | Human | Bg_A20 |
| JAASAAM1097 | *Borrelia garinii* | 2019 | Asia | Tick | Bg_A21 |
| PHei | *Borrelia garinii* | 1987 | Europe | Human | Bg_A22 |
| PMek | *Borrelia garinii* | 1992 | Europe | Human | Bg_A22 |
| PUI | *Borrelia garinii* | 1999 | Europe | Human | Bg_A22 |
| 20047 | *Borrelia garinii* | - | Europe | Tick | Bg_A23 |
| JAASAAM1087 | *Borrelia garinii* | 2019 | Asia | Tick | Bg_A23 |
| JAASAAM1063 | *Borrelia garinii* | 2019 | Asia | Tick | Bg_A24 |
| GESTAN298 | *Borrelia garinii* | 2018 | Europe | Tick | Bg_A25 |
| PLi | *Borrelia garinii* | 1988 | Europe | Tick | Bg_A25 |
| Malouvrh | *Borrelia garinii* | - | Europe | Human | Bg_A26 |
| PKi | *Borrelia garinii* | 1992 | Europe | Human | Bg_A26 |
| PNov | *Borrelia garinii* | 1990 | Europe | Human | Bg_A26 |
| PLa | *Borrelia garinii* | 1988 | Europe | Human | Bg_A27 |

**Table S4.** Output of aBSREL (Smith et al., 2015) analysis from the package HyPhy (https://www.hyphy.org/) run on full PFam54 gene phylogeny reconstructed in MrBayes v. 3.2.6 (Huelsenbeck & Ronquist, 2001; Ronquist et al., 2012) with ploidy set to haploid and a codon substitution model with inverse gamma distributed rate variation, the universal genetic code, and assuming equal selection (ω) (Goldman & Yang, 1994; Muse & Gaut, 1994). In total, 44 branches were chosen for testing based on the following criteria: a branch separating genospecies which utilize different reservoir hosts (bird vs. rodent), branch separating isolates arising from different tick transmission cycles (*I. persulcatus* or *I. ricinus*), or genes known to encode proteins that have been shown to provide protection from host-specific, complement-mediated killing ZQA68 (CspA), BGA66, BGA71, PKO2068 (BaCRASP-1) (Hammerschmidt et al., 2014, 2016; T. Hart et al., 2018; T. M. Hart et al., 2021; Kraiczy, 2016). Branches which showed evidence for diversifying selection are shown in bold. Here B refers to the branch length as imported from MrBayes v. 3.2.6 (Huelsenbeck & Ronquist, 2001; Ronquist et al., 2012), *p*-value referes to the uncorrected *p-*values based on the likelihood ratio test (LRT) performed by aBSREL (Smith et al., 2015).

| **Branch Name** | **B** | **LRT** | **p-value** | **ω_1_** | **ω_1_ (%)** | **ω_2_** | **ω_2_ (%)** |
| --- | --- | --- | --- | --- | --- | --- | --- |
| **N526** | **0.0296** | **26.4981** | **0.0000** | **0.911** | **96%** | **88.3** | **4%** |
| **N588** | **0.0195** | **72.4340** | **0.0000** | **0.222** | **97%** | **264** | **3%** |
| **N597** | **0.5292** | **52.3333** | **0.0000** | **0.492** | **37%** | **9120** | **63%** |
| **N884** | **0.0940** | **41.1664** | **0.0000** | **1.00** | **89%** | **9090** | **11%** |
| **PBi-BGA63** | **0.0211** | **24.1108** | **0.0000** | **0.33** | **99%** | **9050** | **1%** |
| **N905** | **0.0440** | **24.6725** | **0.0000** | **0.00** | **91%** | **119** | **9%** |
| **N544** | **0.0403** | **19.3848** | **0.0000** | **0.321** | **97%** | **3850** | **3%** |
| **N850** | **0.2381** | **17.0506** | **0.0001** | **0.00** | **68%** | **100000** | **32%** |
| **20047-ZQA68** | **0.0065** | **13.9413** | **0.0003** | **0.00** | **98%** | **100000** | **2%** |
| **ZQ1-ZQA73** | **0.2417** | **13.1469** | **0.0005** | **1.00** | **73%** | **100000** | **27%** |
| **N683** | **0.1072** | **11.9452** | **0.0009** | **0.944** | **91%** | **23.1** | **9%** |
| Arh923-BGA68 | 0.0119 | 0 | 1 | 0.239 | 100% | NA | NA |
| J15-BGA68 | 0.0294 | 0 | 1 | 0.269 | 100% | NA | NA |
| PBi-BGA68 | 0.0046 | 0 | 1 | 0.609 | 100% | NA | NA |
| PBi-BGA71 | 0.0216 | -2.5564 | 1 | 1.92 | 100% | NA | NA |
| n524 | 0.2079 | -3.3222 | 1 | 10000000000 | 100% | NA | NA |
| n596 | 0.005 | -3.2658 | 1 | 1.39 | 100% | NA | NA |
| n603 | 0.022 | 0 | 1 | 0.867 | 100% | NA | NA |
| n604 | 0.0133 | 0 | 1 | 0.787 | 100% | NA | NA |
| n615 | 0.0018 | -2.8742 | 1 | 10000000000 | 100% | NA | NA |
| n633 | 0.0024 | 1.042 | 1 | 10000000000 | 100% | NA | NA |
| n644 | 0.0124 | 4.592 | 1 | 0.00 | 93% | 48.6 | 7% |
| n654 | 0.023 | 0.4671 | 1 | 0.00 | 82% | 7.66 | 18% |
| n665 | 0.0067 | -0.8211 | 1 | 10000000000 | 100% | NA | NA |
| n666 | 0.0025 | -2.4882 | 1 | 10000000000 | 100% | NA | NA |
| n670 | 0.0298 | 0 | 1 | 0.302 | 100% | NA | NA |
| n697 | 0.0058 | -2.4554 | 1 | 10000000000 | 100% | NA | NA |
| n705 | 0.0402 | 1.7818 | 1 | 0.000432 | 87% | 8.03 | 13% |
| n714 | 0.0454 | 2.5307 | 1 | 0.993 | 97% | 9090 | 3% |
| n718 | 0.0021 | 0 | 1 | 0.00 | 100% | NA | NA |
| n731 | 0.0066 | 2.3245 | 1 | 0.129 | 99% | 168 | 1% |
| n747 | 0.0112 | 0 | 1 | 0.891 | 100% | NA | NA |
| n764 | 0.0088 | 0 | 1 | 0.612 | 100% | NA | NA |
| n785 | 0.0052 | -2.8659 | 1 | 10000000000 | 100% | NA | NA |
| n788 | 0.0202 | 0 | 1 | 0.641 | 100% | NA | NA |
| n790 | 0.0222 | 0 | 1 | 0.279 | 100% | NA | NA |
| n810 | 0.0241 | 3.6028 | 1 | 10000000000 | 100% | NA | NA |
| n811 | 0.0136 | 0 | 1 | 0.792 | 100% | NA | NA |
| n817 | 0.031 | -2.8862 | 1 | 1.50 | 100% | NA | NA |
| n846 | 0.0025 | 0 | 1 | 0.00 | 100% | NA | NA |
| n854 | 0.02 | -3.3647 | 1 | 1.00 | 100% | NA | NA |
| n867 | 0.0224 | 0 | 1 | 0.000177 | 99.9% | 0.271 | 0.10% |
| n878 | 0.0079 | 0 | 1 | 0.494 | 100% | NA | NA |
| n894 | 0.0167 | -2.5822 | 1 | 1.91 | 100% | NA | NA |


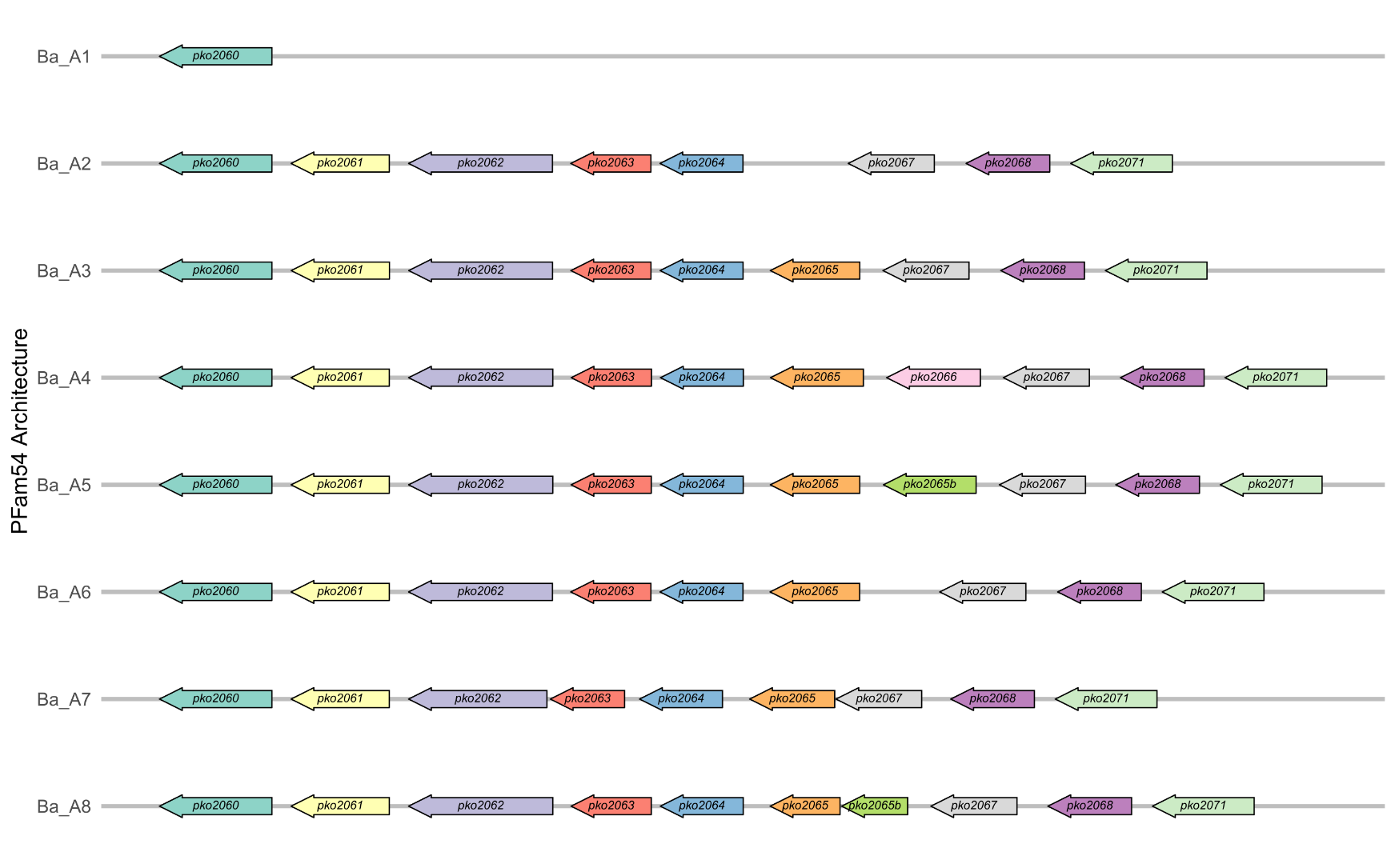
**Figure S1.** Schematic overview of the *Borrelia afzelii* PFam54 gene array architecture types identified in the Eurasian isolates (n=36).


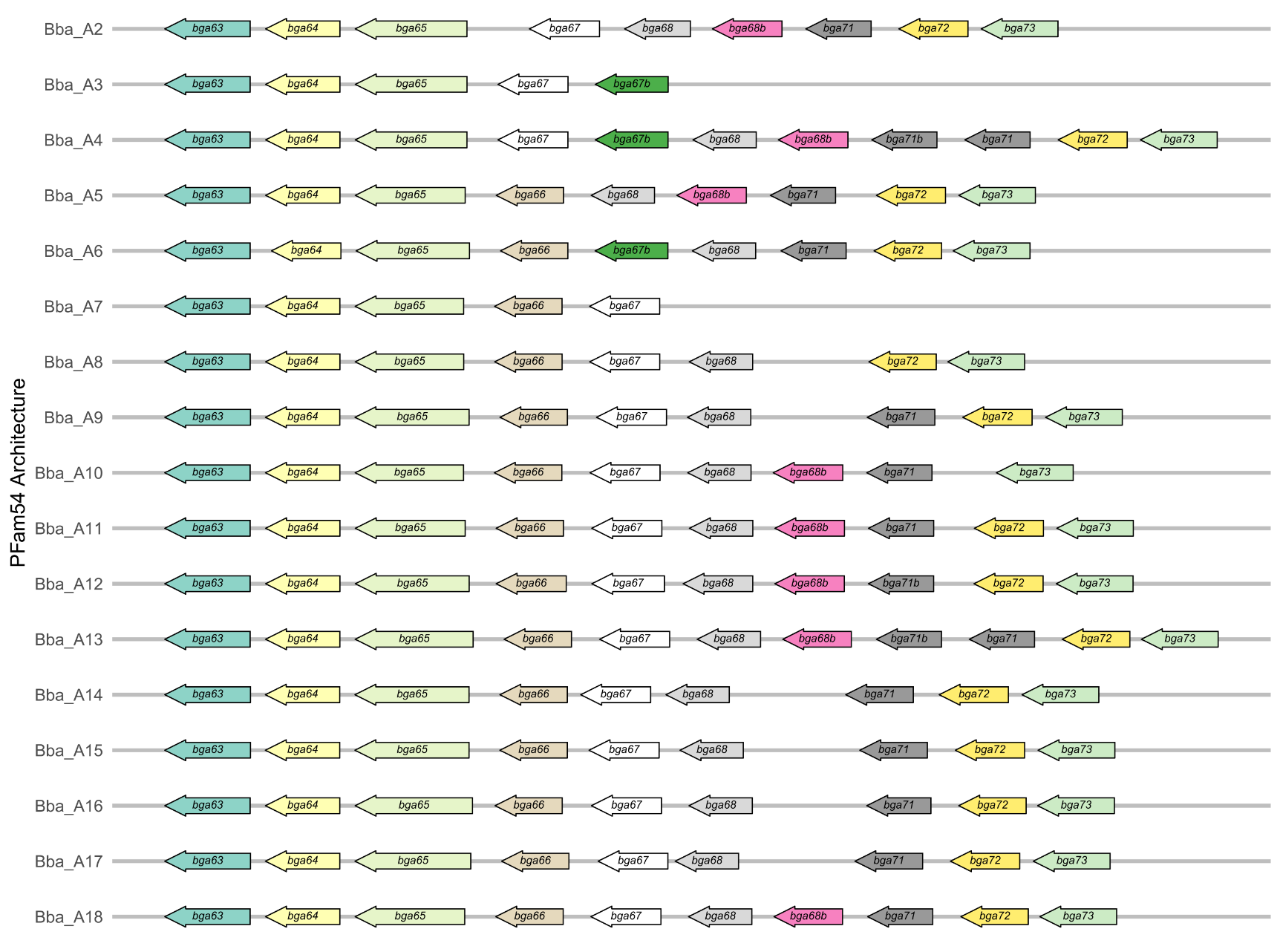
**Figure S2.** Schematic overview of the *Borrelia bavariensis* PFam54 gene array architecture types identified in the Eurasian isolates (n=47). Architecture type Bba_A1 is not shown as this represent two isolates (PBN and PNi) which fully lack the PFam54 gene array (see (Rollins et al., 2022) for full characterization).

**Figure S3.** Schematic overview of the *Borrelia garinii* PFam54 gene array architecture types identified in the Eurasian isolates (n=58).


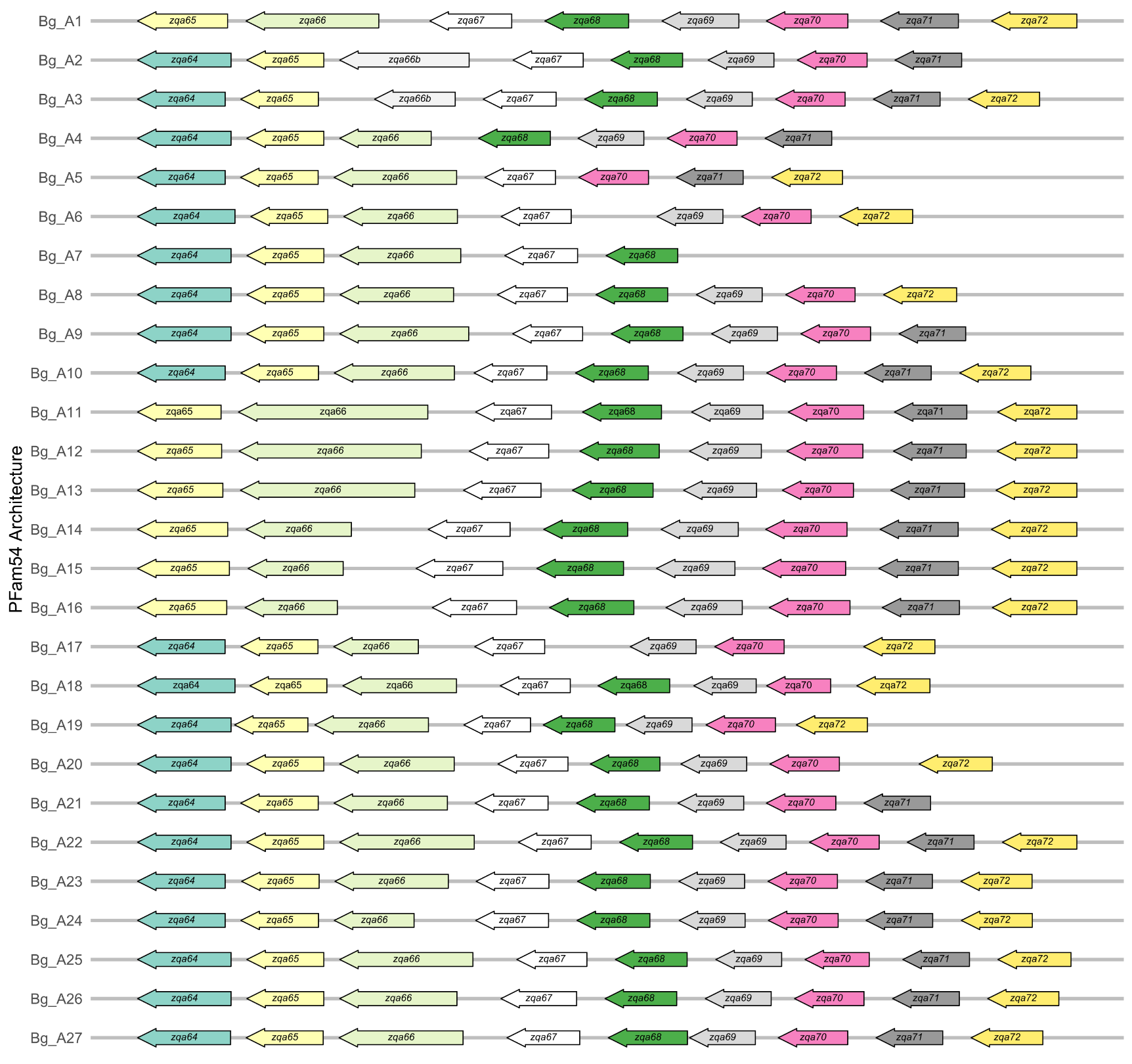


**References**

Goldman, N., & Yang, Z. (1994). A codon-based model of nucleotide substitution for protein-coding DNA sequences. *Molecular Biology and Evolution*, *11*(5), 725–736. https://doi.org/10.1093/oxfordjournals.molbev.a040153

Hammerschmidt, C., Klevenhaus, Y., Koenigs, A., Hallström, T., Fingerle, V., Skerka, C., Pos, K. M., Zipfel, P. F., Wallich, R., & Kraiczy, P. (2016). BGA66 and BGA71 facilitate complement resistance of *Borrelia bavariensis* by inhibiting assembly of the membrane attack complex. *Molecular Microbiology*, *99*(2), 407–424. https://doi.org/10.1111/mmi.13239

Hammerschmidt, C., Koenigs, A., Siegel, C., Hallström, T., Skerka, C., Wallich, R., Zipfel, P. F., & Kraiczy, P. (2014). Versatile roles of CspA orthologs in complement inactivation of serum-resistant Lyme disease spirochetes. *Infection and Immunity*, *82*(1), 380–392. https://doi.org/10.1128/IAI.01094-13

Hart, T. M., Dupuis, A. P., Tufts, D. M., Blom, A. M., Starkey, S. R., Rego, R. O. M., Ram, S., Kraiczy, P., Kramer, L. D., Diuk-Wasser, M. A., Kolokotronis, S. O., & Lin, Y. P. (2021). Host tropism determination by convergent evolution of immunological evasion in the Lyme disease system. *PLoS Pathogens*, *17*(7), 1–29. https://doi.org/10.1371/journal.ppat.1009801

Hart, T., Thien, N., Nguyen, T., Nowak, N. A., Zhang, F., Linhardt, J., Diuk-wasser, M., Ram, S., Kraiczy, P., & Lin, Y. (2018). Polymorphic factor H-binding activity of CspA protects Lyme borreliae from the host complement in feeding ticks to facilitate tick- to-host transmission. *PLoS Pathogens*, *14*(5), e1007105.

Huelsenbeck, J. P., & Ronquist, F. (2001). MRBAYES: Bayesian inference of phylogenetic trees. *Bioinformatics*, *17*(8), 754–755. https://doi.org/10.1093/bioinformatics/17.8.754

Kraiczy, P. (2016). Hide and seek: How Lyme disease spirochetes overcome complement attack. *Frontiers in Immunology*, *7*(SEP). https://doi.org/10.3389/fimmu.2016.00385

Muse, S. v, & Gaut, B. S. (1994). A likelihood approach for comparing synonymous and nonsynonymous nucleotide substitution rates, with application to the chloroplast genome. *Molecular Biology and Evolution*, *11*(5), 715–724. https://doi.org/10.1093/oxfordjournals.molbev.a040152

Rollins, R. E., Wülbern, J., Röttgerding, F., Nowak, T. A., Hepner, S., Fingerle, V., Margos, G., Lin, Y.-P., Kraiczy, P., & Becker, N. S. (2022). Utilizing Two *Borrelia bavariensis* Isolates Naturally Lacking the PFam54 Gene Array To Elucidate the Roles of PFam54-Encoded Proteins. *Applied and Environmental Microbiology*, *88*(5), e01555-21.

Ronquist, F., Teslenko, M., Van Der Mark, P., Ayres, D. L., Darling, A., Höhna, S., Larget, B., Liu, L., Suchard, M. A., & Huelsenbeck, J. P. (2012). MrBayes 3.2: Efficient bayesian phylogenetic inference and model choice across a large model space. *Systematic Biology*, *61*(3), 539–542. https://doi.org/10.1093/sysbio/sys029

Smith, M. D., Wertheim, J. O., Weaver, S., Murrell, B., Scheffler, K., & Kosakovsky Pond, S. L. (2015). Less Is More: An Adaptive Branch-Site Random Effects Model for Efficient Detection of Episodic Diversifying Selection. *Molecular Biology and Evolution*, *32*(5), 1342–1353. https://doi.org/10.1093/molbev/msv022
